# Formalin-Fixed Paraffin-Embedded Tissues—An Untapped Biospecimen for Biomonitoring DNA Adducts by Mass Spectrometry

**DOI:** 10.3390/toxics6020030

**Published:** 2018-06-01

**Authors:** Byeong Hwa Yun, Jingshu Guo, Robert J. Turesky

**Affiliations:** Masonic Cancer Center and Department of Medicinal Chemistry, University of Minnesota, 2231 6th St. SE, Minneapolis, MN 55455, USA; bhyun@umn.edu (B.H.Y.); guoj@umn.edu (J.G.)

**Keywords:** carcinogen, DNA adducts, biomonitoring, formalin-fixed paraffin-embedded tissues, biomarker, mass spectrometry

## Abstract

The measurement of DNA adducts provides important information about human exposure to genotoxic chemicals and can be employed to elucidate mechanisms of DNA damage and repair. DNA adducts can serve as biomarkers for interspecies comparisons of the biologically effective dose of procarcinogens and permit extrapolation of genotoxicity data from animal studies for human risk assessment. One major challenge in DNA adduct biomarker research is the paucity of fresh frozen biopsy samples available for study. However, archived formalin-fixed paraffin-embedded (FFPE) tissues with clinical diagnosis of disease are often available. We have established robust methods to recover DNA free of crosslinks from FFPE tissues under mild conditions which permit quantitative measurements of DNA adducts by liquid chromatography-mass spectrometry. The technology is versatile and can be employed to screen for DNA adducts formed with a wide range of environmental and dietary carcinogens, some of which were retrieved from section-cuts of FFPE blocks stored at ambient temperature for up to nine years. The ability to retrospectively analyze FFPE tissues for DNA adducts for which there is clinical diagnosis of disease opens a previously untapped source of biospecimens for molecular epidemiology studies that seek to assess the causal role of environmental chemicals in cancer etiology.

## 1. Metabolism, Bioactivation, and DNA Adducts as Biomarkers of Exposure and Health Risk

### 1.1. Xenobiotic Metabolism and Bioactivation of Procarcinogens

Humans are continuously exposed to potentially hazardous chemicals in the environment, diet, medicines, and through occupational exposures. Many of these chemicals undergo biotransformation by phase I and/or phase II enzymes to produce reactive electrophiles that can form adducts with macromolecules [1]. Cytochrome P450s (P450s) are by far the most important Phase I enzymes involved in xenobiotic metabolism [2]. P450s catalyze a variety of reactions, including aliphatic and aromatic hydroxylation, *N*- or *O*-dealkylation, aliphatic desaturation, hetero atom oxidation, and epoxidation reactions [2]. The resulting metabolites can contain functional groups such as –OH, –NH_2_, and –SH which can undergo conjugation reactions by phase II enzymes, including UDP-glucuronosyltransferases (UGTs), sulfotransferases (SULTs), *N*-acetyltransferases (NATs), glutathione *S*-transferases (GSTs), and methyltransferases [3].

**Figure 1:**
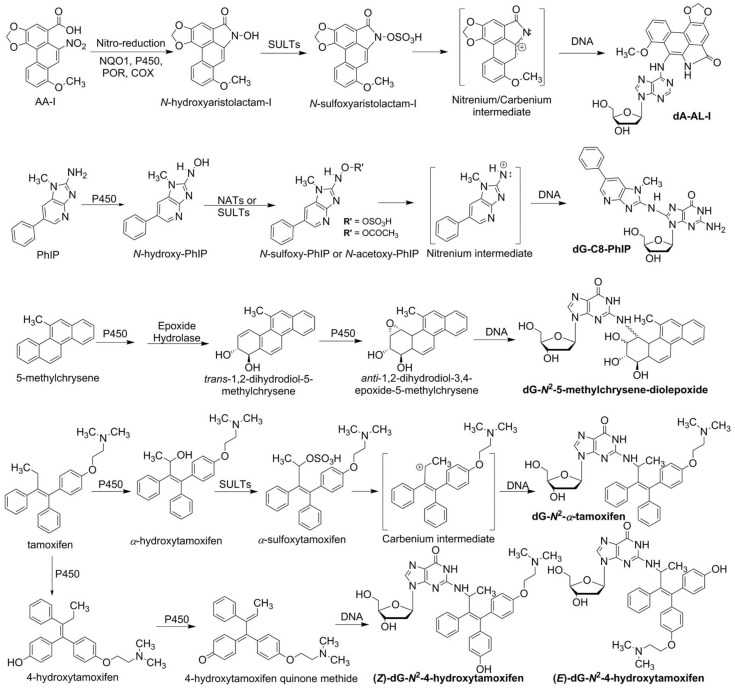
The metabolic activation of aristolochic acid I (AA-I), 2-amino-1-methyl-6-phenylimidazo[4,5-*b*]pyridine (PhIP), 5-methylchrysene, and tamoxifen are shown as prototypes of procarcinogens. Bioactivation is carried out with phase I and/or phase II enzymes, which lead to the formation of DNA adducts. AA-I undergoes nitro-reduction through NAD(P)H:quinone oxidoreductase (NQO1), cytochrome P450s 1A1 and 1A2, NADPH:P450 reductase (POR) or prostaglandin H synthase (COX). The resulting *N*-hydroxyaristolactam-I is bioactivated by SULTs to form an unstable *N*-sulfoxy ester, which quickly undergoes heterolytic cleavage to produce the reactive nitrenium/carbenium intermediate that forms dA-AL-I and other DNA adducts. PhIP undergoes *N*-hydroxylation by P450s, then it is further bioactivated by NATs or SULTs to form *N*-acetoxy or *N*-sulfoxy esters, which lead to the formation of dG-C8-PhIP through the nitrenium intermediate. 5-Methylchrysene undergoes epoxidation (P450s 1A1 and 1B1) followed by epoxide hydroxylation (epoxide hydrolase) on the bay-region phenyl ring, to form the corresponding *trans*-1,2-dihydrodiol-5-methylchrysene. A subsequent round of monooxygenation leads to the formation of *anti*-1,2-dihydrodiol-3,4-epoxide-5-methylchrysene, which can form a DNA adduct at the *N*^2^-atom of dG (dG-*N*^2^-5-methylchrysene-diolepoxide). Two pathways are involved in the DNA adduct formation of the bioactivated tamoxifen. In the first pathway, oxidation of the allylic ethyl side chain results in the formation of *α*-hydroxytamoxifen. The subsequent esterification catalyzed by SULTs leads to the reactive carbenium intermediate and the dG-*N*^2^-α-hydroxytamoxifen adduct. The second pathway involves aryl-oxidation of one of the phenyl rings to yield 4-hydroxytamoxifen quinone methide, a reactive electrophile that can form the DNA adducts. Both pathways lead to (*Z*)- or (*E*)-dG-*N*^2^-4-hydroxytamoxifen.

While many Phase I metabolites are detoxification products, some oxidative metabolites are reactive electrophiles, which can induce toxicity or genotoxicity by covalently binding to protein or DNA, or generate free radicals that deplete cellular antioxidants and induce oxidative stress [4,5]. In a similar vein, many phase II enzyme reactions are regarded as detoxification pathways, and the resulting metabolites are efficiently eliminated from the body. However, in some cases, reactive intermediates are generated, and the metabolites can bind to proteins and DNA. The *O*-acetylation or *O*-sulfation of aromatic amines and heterocyclic aromatic hydroxylamines [6], glutathione conjugation of ethylene dibromide [7], *O*-sulfation of hydroxymethyl polycyclic aromatic hydrocarbons [8], and the acyl glucuronidation of carboxylic acid moieties of nonsteroidal anti-inflammatory drugs (NSAIDs) [9] are examples of conjugation reactions leading to reactive intermediates. The metabolic activation of rodent and possible human carcinogens including 2-amino-1-methyl-6-phenylimidazo[4,5-*b*]pyridine (PhIP) [10], aristolochic acid I (AA-I) [11], 5-methylchrysene [12,13] and tamoxifen [14,15], are shown as examples of procarcinogens that require phase I and/or II enzymes to produce penultimate species that bind to DNA (Figure 1).

Rodents are often employed as experimental laboratory animals to study metabolism of hazardous chemicals, to screen for DNA adduct formation, and elucidate mechanisms of carcinogenesis [5]. The metabolism of carcinogens and their biological effects in animal models can differ from humans because of species differences in catalytic activities of phase I and II enzymes involved in bioactivation or detoxification [10,16,17,18]. Thus, animal carcinogen bioassay data may not accurately gauge health risk of some chemicals in humans. However, DNA adducts of carcinogens, which are measures of the biologically effective dose, can serve as biomarkers for the extrapolation of genotoxicity data from animal studies for human risk assessment [19,20].

Epidemiological studies have reported that exposures to different chemicals in the diet and environment, or lifestyle factors, such as tobacco usage and alcohol consumption, are linked to the increased risk of developing certain types of cancers. As examples, polycyclic aromatic hydrocarbons (PAHs) in cigarette smoke are linked to lung cancer [21]; occupational exposures to aromatic amines are linked to bladder cancer [21,22]; usage of traditional Chinese herbal medicines containing AA-I are linked to upper urothelial cancer [23,24]; and consumption of aflatoxin B_1_ (AFB_1_) produced by fungi on agricultural crops, is a risk factor for liver cancer [25,26]. 

The identification and quantitation of DNA adducts is a first step in elucidating the potential role of a genotoxic chemical in the etiology of cancer [19,20]. The identification DNA adducts in human tissues are likely to represent a combination of recent and longer-term exposures to certain hazardous chemicals. The interpretation of negative findings, or the absence of DNA adducts, must be done with caution, since many adducts can undergo repair [27]. Ideally, the biomonitoring of DNA adducts should be conducted when the multistage process of tumorigenesis began, rather than many years later when the cancer is diagnosed. However, life-style factors such as tobacco smoking, diet, and environmental pollution often represent long-term exposures, and current adduct levels of carcinogens from these exposures are likely to correlate with adduct levels that existed during the time of tumor initiation and progression. 

### 1.2. Methods to Measure DNA Adducts

The measurement of DNA adducts in humans is a challenging analytical task because the levels of DNA adducts generally occur at less than one adduct per 10^7^ nucleotides, and the amount of tissue available for measurement is limited. Even for blood, a readily accessible biofluid, the amount of DNA obtained is usually a few up to several tens of micrograms scale. Thus, highly sensitive and specific methods are required to measure DNA adducts in humans. During the past three decades, the major techniques employed to measure DNA adducts have been ^32^P-postlabeling [28,29], antibody-based immunoassay/immunohistochemistry (IHC) [30,31], gas chromatography-mass spectrometry (GC-MS) [32], and most recently, liquid chromatography-mass spectrometry (LC-MS) [33,34,35,36,37].

^32^P-postlabeling is a highly sensitive method to detect DNA adducts. The DNA is enzymatically digested to 3′-phospho-2′-deoxyribonucleotides, and ^32^P-orthophosphate from [γ-^32^P] ATP is transferred to the 5′-OH position of the 2′-deoxyribonucleotide adduct, by polynucleotide kinase. The adducted 5′-^32^P-labeled nucleotides are resolved by multi-dimensional thin-layer chromatography with polyethylenimine-modified cellulose plate, or by polyacrylamide electrophoresis, using autoradiography for detection, or by HPLC with radiometric detection [28,29,38,39]. The assay only requires 1–10 µg of DNA, and the sensitivity for some adducts can reach a limit of detection as low as one adduct per 10^10^ nucleotides [29]. Studies in rodents and humans employing ^32^P-postlabeling methods have shown that many genotoxic chemicals undergo metabolism and covalently adduct to DNA in many organs [29,40,41]. However, there are several limitations of the ^32^P-postlabeling assay. The technique is labor intensive and its usage requires large amounts of hazardous phosphorous radioactivity. Moreover, the technique is not quantitative [42], and structural information about the identity of the adduct is uncertain, particularly in humans where many overlapping lesions are present [29,40]. Thus, epidemiology studies employing ^32^P-postlabeling often provide equivocal data about chemical exposures linked to DNA adducts and cancer risk [43,44,45,46]. 

Immunodetection relies on the generation of monoclonal or polyclonal antibodies raised against modified-DNA adducts coupled to carrier proteins, or carcinogen-treated DNA, where usually very high levels of modification, about one modified base to 100 nucleotides, are required for successful generation of a titer [30,47]. The sensitivity of the method depends on the affinity of the antibody, but a detection limit of about one adduct per 10^8^ nucleotides for certain DNA adducts can be reached, when detected by fluorescence or chemiluminescence spectroscopy [48,49]. IHC detection of DNA adducts in tissue section-cuts mounted on slides is generally less sensitive than immunoassays performed on isolated DNA; however, IHC allows the visualization of the DNA adduct within specific cell types of a tissue, and is especially suitable for archived human formalin-fixed paraff in-embedded (FFPE) tissues (Section 3) [50]. Cross-reaction of the antibody with DNA adducts of similar structure or cellular components can occur [30,31], which raises concerns about the specificity of the methodology. Immunodetection methods have made significant contributions to the biomonitoring of DNA adducts; however, similarly to the ^32^P-postlabeling method, immunodetection does not provide structural information to confirm adduct identity, and the method is semi-quantitative. 

GC-MS with electron impact ionization, and more recently, negative ion chemical ionization has been employed to measure DNA adducts (primarily used for oxidized DNA bases) where adduct structures can be corroborated from the MS fragmentation spectra [32]. Often, the DNA is hydrolyzed with formic acid or by elevated temperature under neutral pH conditions. Most DNA adducts require chemical derivatization to increase the volatility required for GC analysis. The derivatization process can complicate the analysis and introduce artifact formation, particularly for oxidized DNA base measurements [51]. In contrast, the online coupling of capillary electrophoresis or LC to electrospray ionization (ESI) MS is a breakthrough technology that can measure many DNA adducts which would otherwise undergo thermal decomposition by GC-MS [52]. 

Currently, LC-ESI-multistage MS (MS^n^) is the predominant platform for DNA adduct analyses [33,35,37,53]. The rapidly advancing technologies in LC-MS instrumentation have attained ultra-high sensitivity and selectivity, particularly with ion trap and high resolution accurate mass spectrometry (HRAMS). These platforms include the coupling of nano-flow chromatography and nanoESI source, and versatile and flexible scanning strategies. The detection of DNA adducts at levels as low as one per 10^11^ nucleotides have been reported using a hybrid Orbitrap MS [54]. Both targeted and non-targeted MS scan approaches have been employed to identify many DNA chemical modifications [35,36,37,55,56,57,58].

**Figure 2:**
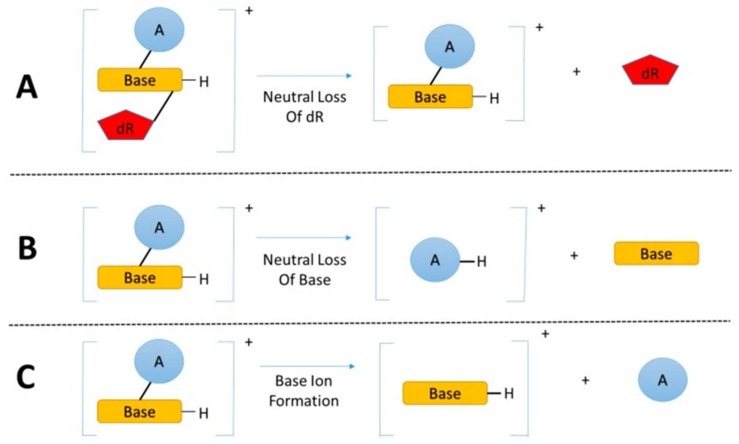
The fragmentation pathways of modified nucleosides analyzed by LC-MS. (**A**) The major fragmentation of the modified nucleosides is the neutral loss of deoxyribose. Other common fragmentations include (**B**) the neutral loss of base and (**C**) the neutral loss of the adduct with the formation of base ions [58].

The DNA is typically digested with a cocktail of nucleases prior to adduct measurements by LC-MS. The digestion products contain adducts formed at the DNA bases of the 2′-deoxyribonucleosides, or in rarer cases, adducts are formed at the phosphate backbones [59,60]. A common feature for many DNA adducts is their tendency to lose the deoxyribose moiety (dR, 116 or 116.0473 Da in HRAMS), when subjected to collision-induced dissociation (CID) [61]. The transition between the adduct precursors ([M + H]^+^) and their aglycones after losing dR ([M + H − 116]^+^) is commonly targeted to detect and quantify DNA adducts in MS^n^. The constant neutral loss of molecules, such as dR from the 2′-deoxyribonucleosides, serves as the foundation of the “DNA adductomics” approach [37,55,56,62,63]. Figure 2 shows the fragmentation pathways of modified nucleosides, where the major ions are the chemically modified bases after neutral loss of dR, or in less frequent cases the bases are eliminated as the neutral fragment and the carcinogen moieties retain the charge. These types of MS transitions are usually monitored in the targeted and un-targeted approaches by LC-MS [58,63]. 

## 2. Overview and the History of Formalin Fixation Process

While great strides have been made in the detection of DNA adducts in humans, fresh tissues obtained from biopsies or post-mortem are often not available. The paucity of fresh tissue specimens has hampered the advancement of DNA adduct biomonitoring in human studies. However, archived FFPE tissue specimens with clinical diagnosis of disease are a largely untapped biospecimen and often available for DNA adduct biomarker research.

**Figure 3:**
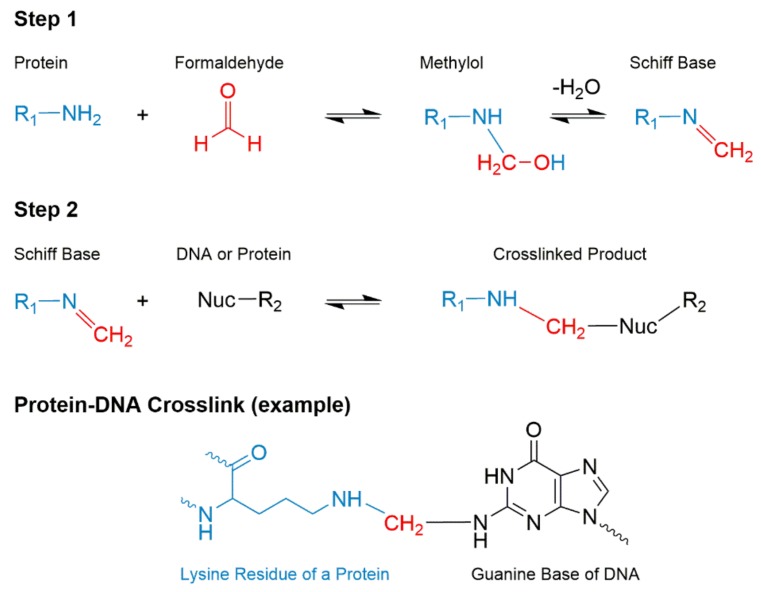
The reactions of formaldehyde mediated crosslinking of DNA and protein. Formaldehyde diffuses through tissue and reacts with a nucleophilic sites of protein and/or DNA base resulting in unstable intermediates of methylol and Schiff base. Then, a second nucleophile from inter- or intramolecular DNA or protein attacks the Schiff base resulting in a crosslinked complex. A specific example of a protein-DNA crosslink is shown. The atoms are color coded: *cyan*, protein; *red*, formaldehyde; and *black*, DNA. Reproduced with permission from [65]. Copyright ASBMB, 2015.

Formalin, 10% neutral buffered formaldehyde solution, is the most commonly used fixative worldwide [64]. During the process of formalin fixation, formaldehyde undergoes multiple steps of reactions with cellular nucleophilic species to form molecular crosslinks [64,65]. Formaldehyde permeates through the tissue, and the nucleophilic moieties of amino acids and nucleobases attack the formaldehyde yielding unstable intermediates of methylol adducts and Schiff bases [65]. These intermediates are stabilized by forming methylene bridges with a second nucleophilic group, often on another molecule. The methylene bridges formed with DNA and protein are stable crosslinks at room temperature (Figure 3); however, the linkages are reversible by heat treatment and/or under alkaline pH [66,67]. The reversal rate of the crosslink increases exponentially as a function of temperature [66]. The efficacy of reversal of formaldehyde-mediated crosslinks is the most critical feature that impacts the quantitative analysis of RNA, DNA, and protein biomarkers in FFPE tissues. 

### Technical Challenges and Breakthrough Technology in DNA Recovery from FFPE Tissues 

FFPE tissues are now widely used in high throughput genomic [64,68,69,70,71], proteomics [72,73,74], and to a lesser extent, metabolomics studies [75,76]. The crucial step in these applications is the quantitative extraction of the molecules of interest. In genomic sequencing studies, the conventional method of DNA isolation from FFPE tissues has often employed elevated temperature (up to 100 °C) and alkaline pH (>9) to achieve a complete reversal of crosslinks. Many automated methods employed in cancer genomics still use elevated temperature to isolate DNA from FFPE tissues. The recovered DNA can serve as a template for PCR amplification. However, these harsh conditions can cause oxidation of nucleobases or depurination of chemically-modified nucleobases, and thus, are not compatible for quantitative measurements of DNA adducts. Moreover, even though formalin-fixation is the most common method of tissue preservation world-wide, the conditions of fixation can vary in different laboratories. A prolonged time of tissue preservation in formalin results in over-fixation of the tissue and leads to inefficient hydrolysis of crosslinks between DNA and protein. Therefore, the yield and quality of the recovered DNA is decreased [77,78,79]. Thus, the development of robust analytical methods to quantitatively recover DNA adducts from FFPE tissue has been a challenging endeavor.

## 3. Measurement of DNA Adducts in FFPE Tissues by IHC, ^32^P-Postlabeling, and LC-MS

### 3.1. IHC Detection of DNA Adducts

FFPE specimens are often used for immunodetection of DNA adducts, most commonly by IHC methods [30,80]. In contrast to mass spectrometry-based methods, which break down the DNA to the mono 2′-deoxyribonucleoside or DNA base (*vide supra*), IHC methods employ intact DNA. The detection of DNA damage can be carried out on either fixed cells such as lymphocytes, exfoliated oral or bladder cells, or with FFPE tissue section-cuts. The cells or FFPE tissue section-cuts are mounted on glass slides for IHC analysis. Procedures are often used to increase the accessibility of the antibody to the carcinogen DNA adduct to increase the sensitivity of the assay. These procedures can include treatment with proteases to remove histone and other proteins from the DNA, followed by treatment with RNase to eliminate potential cross-reactivity with RNA adducts. Mild acid or base treatment also may be performed to denature the DNA and further increase the accessibility of the antibody to the adduct. It is imperative that the adduct is stable to the denaturing treatment conditions for validation of the IHC technique. The two most commonly used detection systems for visualization of DNA adduct-antibody complexes are immunofluorescence or chromophores, where the secondary antibody is tagged with a chemically conjugated fluorophore, a peroxidase or alkaline phosphatase enzyme. [30,81].

Table 1 summarizes examples of IHC detection of DNA adducts in FFPE tissues. Santella’s group detected and quantified DNA adducts of 4-aminobiphenyl (4-ABP), an aromatic amine and a human bladder carcinogen that is formed in tobacco smoke [21,22], and also occurs as a contaminant in some commercial hair dyes [82]. 4-ABP-adducted DNA was detected in uroepithelium of bladder cancer patients [83]. The level of the 4-ABP adduct was correlated with the smoking status and *p53* overexpression, a response to DNA damage. There was linear relationship between the relative degree of DNA adduct staining and the number of cigarettes smoked. The same group also detected DNA adducts of polycyclic aromatic hydrocarbons (PAHs) in archived breast tissues sections using polyclonal antiserum [84,85]. PAHs are incomplete combustion products of organic matter and found in cereal and grain products, some oils, and also found in charred meat and tobacco smoke [42]. PAHs have been linked to human cancers at multiple sites [21]. The most well studied PAH is benzo[*a*]pyrene (B[*a*]P), a human lung carcinogen found as an environmental pollutant, and it also occurs in tobacco smoke, and charred meat [42,86]. The Poirier laboratory developed an antibody raised against DNA modified with *r*7,*t*8-dihydoxy-*t*-9,10-oxy-7,8,9,10-tetrahydrobenzo[*a*]pyrene (BPDE) [87], which later was shown to cross-react with other structurally similar diol-epoxide-PAH-DNA adducts [88]. This PAH-DNA antiserum has been used to screen for DNA adducts in FFPE tissues from human esophagus [81,89], prostate [90], cervix [91], vulva [47], and placenta [80]. A significantly higher level of staining of presumed B[*a*]P adducts was found in benign breast disease in comparison to the cancerous tissues of patients, possibly due to cellular proliferation and dilution of the adduct in cancerous tissue [84,85]. Rundle et al. employed IHC to measure PAH-DNA adducts and examined the associations with alcohol consumption and the influence of GSTM1 genotype on DNA adduct formation in FFPE breast tissues [92]. Subjects harboring the GSTM1-null genotype, which lacks the expression of GTSM1, an enzyme that detoxicates PAH diol-epoxides [93], had increased levels of DNA adducts among current alcohol consumers, but not among nondrinkers. In contrast, in benign tissues from controls, no association was observed between genotype and adduct levels, regardless of drinking status. Poirier also analyzed tamoxifen-DNA adducts in rat hepatocytes by IHC [94]. A steady increase in adduct levels was observed with chronic exposure. 

Shirai et al. developed polyclonal antibodies against DNA adducts of 3,2′-dimethyl-4-aminobiphenyl (DMAB), an aromatic amine that induces tumors at multiple sites in rodent models, and PhIP, a probable human carcinogen formed in cooked meat that induces tumors in colorectum and prostate of rodents [95,96,97]. Dose-related nuclear staining was observed in various acetone-fixed tissues of rodents 24 h after single exposure of DMAB or PhIP. Using the same polyclonal serum, putative DNA adducts of PhIP were detected, by IHC, at high frequency in mammary tissue of women with breast cancer [98] and in prostate tissue of men with prostate cancer [99]. However, these results are at odds with specific mass spectrometry-based methods, where PhIP DNA adducts were detected at considerably lower frequency and at much lower levels of DNA modification in both tissues [100,101]. The discrepancy between the estimates of the PhIP DNA adduct reported by MS and IHC methods suggest the possible cross-reactivity of the polyclonal antibodies with other DNA adducts of similar structure or endogenous cellular components. There is a need to cross-corroborate the identities and levels of DNA adducts measured by IHC and specific MS-based methods. Aoshiba and coworkers raised antibodies against 8-hydroxy-2′-deoxyguanosine (8-OHdG), an oxidative DNA adduct, and 4-hydroxy-2-nonenal (4-HNE), a lipid peroxidation adduct, to evaluate the oxidative stress induced by cigarette smoke in paraffin-embedded pulmonary epithelial cells of mice [102]. There was a dramatic increase in the intensity of signals one hour post cigarette smoke exposure, compared to pre-exposure, which confirmed the causal role of cigarette smoking in oxidative damage to respiratory epithelium. 

### 3.2. DNA Measurements in FFPE Tissues by ^32^P-Postlabeling

There is only one report employing ^32^P-postlabeling to detect DNA adducts in FFPE tissues [103]. In that study, rat tissues were fixed in formalin and embedded in paraffin after dosing with B[*a*]P or 2-acetylaminofluorene (2-AAF). DNA was extracted from fixed tissues using a modified phenol-chloroform method [106]. The levels of DNA adduct recovered from FFPE tissues were significantly lower than the levels obtained from fresh frozen tissues. The authors concluded that FFPE tissues could be used to screen for DNA adducts but that adduct levels may be underestimated particularly with prolonged time of fixation in formalin. 

### 3.3. Measurement of DNA Adducts in FFPE Tissues of Rodents and Human by LC-MS

The physio-chemical data provided by MS for proof of DNA adduct structure combined with the robust quantitation and high sensitivity makes MS the technique of choice for DNA adduct biomarker measurements. The DNA adducts must be stable towards both the formalin fixation and DNA retrieval processes. Furthermore, the DNA must be fully digestible by nucleases to monodeoxyribonucleosides. Until recently, the recovery of high quality DNA completely devoid of formalin crosslinks was difficult to achieve under mild hydrolysis conditions. However, commercial kits from several vendors now employ mild retrieval conditions at neutral pH to reverse the crosslinks of FFPE DNA. The DNA recovered was shown to be successfully employed as templates for amplification by PCR. We tested commercial kits from several vendors and found the FFPE miniprep kit from Zymo Research (Irvine, CA, USA), with some modifications in manufacturer’s protocol, provided high quality DNA that was fully digestible by nucleases [79,101,105]. 

**Figure 4:**
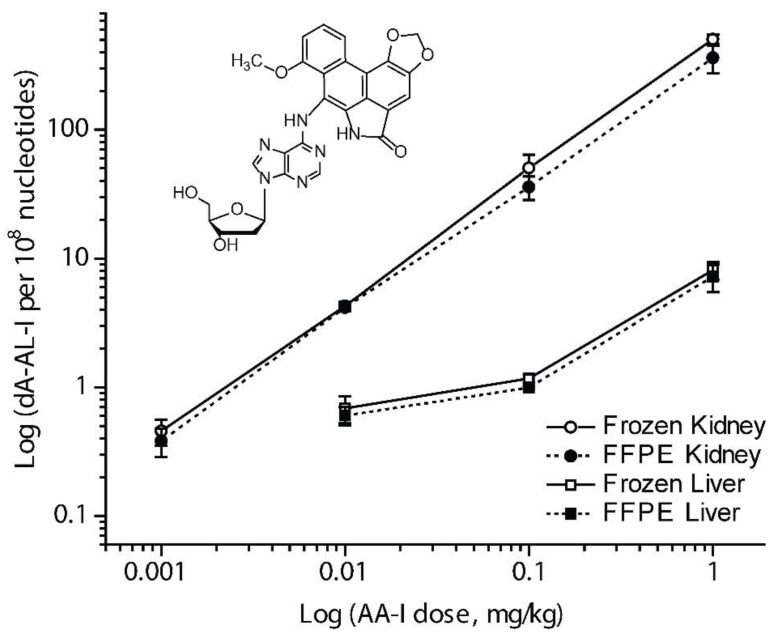
Mean level of dA-AL-I adducts present in mouse kidney and liver following treatment with AA-I (0.001−1 mg/kg body weight). Adduct levels measured in freshly frozen and FFPE mouse kidney (○ and ●) and liver (□ and ■) (mean adduct level, SD, *N* = 4 animals per dose, quadruplicate measurements per animal) were plotted as a function of dose. The overall mean difference in adduct levels between freshly frozen and FFEP kidney and liver tissues across all doses was 21 ± 14% (mean ± SD). dA-AL-I adduct formation was below the limit of detection in liver of mice dosed with AA-I at 0.001 mg/kg body weight. Mean levels of dA-AL-I adducts were significantly statistically different between freshly frozen and FFPE kidney or liver at the following dose treatments of AA-I: kidney, 1 mg/kg, *p* = 0.03; liver, 0.1 mg/kg, *p* = 0.01; unpaired two-tailed *t*-test. Reproduced with permission from [104]. Copyright ACS, 2013.

Our laboratory established a method to isolate DNA from FFPE liver and kidney tissues of C57BL/6J mice, using aristolochic acid I (AA-I) as the model carcinogen [79,104]. AA-I is an upper urinary tract human carcinogen found in Aristolochia plants, some of which have been used in traditional Chinese herbal medicines [79,104]. DNA was isolated from freshly frozen tissue by the phenol-chloroform method, and DNA from FFPE tissue was isolated with the FFPE miniprep kit (Zymo Research). AA-I DNA adducts were measured by ultra-performance liquid chromatography-electrospray ionization-ion trap-multistage MS^n^ scanning (UPLC-ESI-IT-MS^3^). Across all dosing levels, the amounts of AA-I DNA adduct in DNA from FFPE tissues were comparable to those of matching freshly frozen tissues (Figure 4) [104]. 

Then, we examined the effect of duration of formalin fixation on the recovery of DNA and the level of DNA adducts in rodents treated with AA-I [79]. The yield of DNA retrieved from formalin-fixed tissues decreased as a function of fixation time, and only 30% of DNA was recovered from FFPE tissues after one week of fixation in formalin compared to the freshly frozen tissues. However, the DNA retrieved was completely digested by nucleases and the levels of AA-I DNA adduct were relatively constant between the freshly frozen tissues and FFPE tissues. DNA fragments of 184 and 327 bp extracted from FFPE tissues were readily amplified by PCR, and the quality of sequence data was comparable to that obtained from DNA obtained of fresh frozen tissues [79]. Our findings demonstrate that the DNA can be recovered from FFPE tissue to analyze DNA adducts of AA-I in FFPE tissue, and adducts of AA-I or other carcinogens may be correlated with mutational signatures induced in tumor tissue.

**Figure 5:**
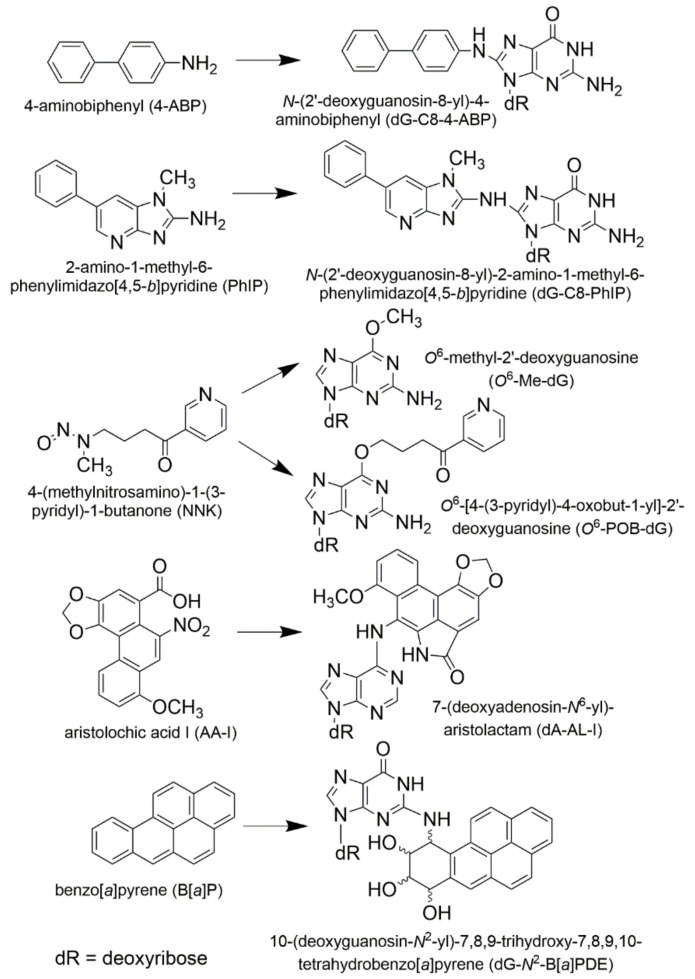
Structures, names, and abbreviations of carcinogens and their adducts used for quantitation of multiclass carcinogenic DNA adducts in freshly frozen and FFPE tissues of rodents. Reproduced with permission from [105]. Copyright ACS, 2016.

Thereafter, we sought to determine if our method of DNA adduct retrieval from FFPE tissues could be employed to measure DNA adducts of other environmental and dietary genotoxicants. We examined DNA adducts of four important classes of environmental and dietary carcinogens: PAHs (B[*a*]P), aromatic amines (4-ABP), HAAs (PhIP), and *N*-nitroso compounds 4-(methylnitrosamino)-1-(3-pyridyl)-1-butanone (NNK), which is found in tobacco and a lung carcinogen [21,107]. The major DNA adducts of these carcinogens studied were: 10-(2′-deoxyguanosin-*N*^2^-yl)-7,8,9-trihydroxy-7,8,9,10-tetrahydrobenzo[*a*]pyrene (dG-*N*^2^-B[*a*]PPDE), *N*-(2′-deoxyguanosin-8-yl)-4-ABP (dG-C8-4-ABP), *N*-(2′-deoxyguanosin-8-yl)-PhIP (dG-C8-PhIP), O6-[4-oxo-4-(3-pyridy)-butyl]-2′-deoxyguanosine (*O*^6^-POB-dG), *O*^6^-methyl-2′-deoxyguanosine (*O*^6^-methyl-dG) (Figure 5) [105]. All of these adducts and dA-AL-I were measured by UPLC-ESI-IT-MS^3^ with the stable isotope dilution method. The levels of DNA adducts in FFPE tissues of rodents preserved in formalin for 24 h were at levels comparable to those levels measured in freshly frozen tissues [105]. 

**Figure 6:**
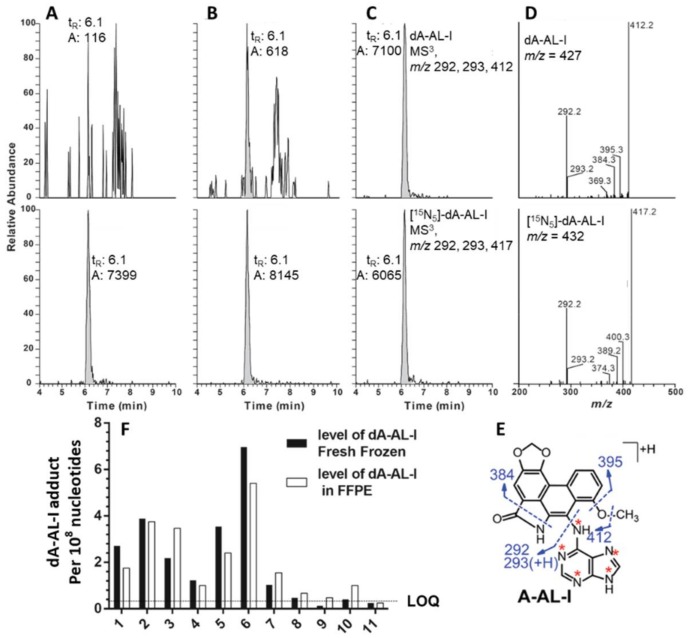
Extracted ion chromatograms of dA-AL-I from human kidney cortex of patients with upper urothelial cancer from Taiwan at levels (**A**) below the LOQ, and positive samples at (**B**) 0.4 adducts, and (**C**) 5.9 adducts per 10^8^ bases. The product ion spectra of dA-AL-I obtained from panel C is depicted in (**D**) along with the internal standard [15N5]-dA-AL-I (E, 15N labels of the internal standard of dA-AL-I are depicted with asterisks). Insert (**F**) dA-AL-I adduct levels in matching fresh frozen and FFPE kidney samples, containing both renal cortex and medulla, obtained from 11 individuals residing in endemic regions of Croatia and Serbia who underwent nephroureterectomy for upper urothelial cancer. Reproduced with permission from [104]. Copyright ACS, 2013.

**Figure 7:**
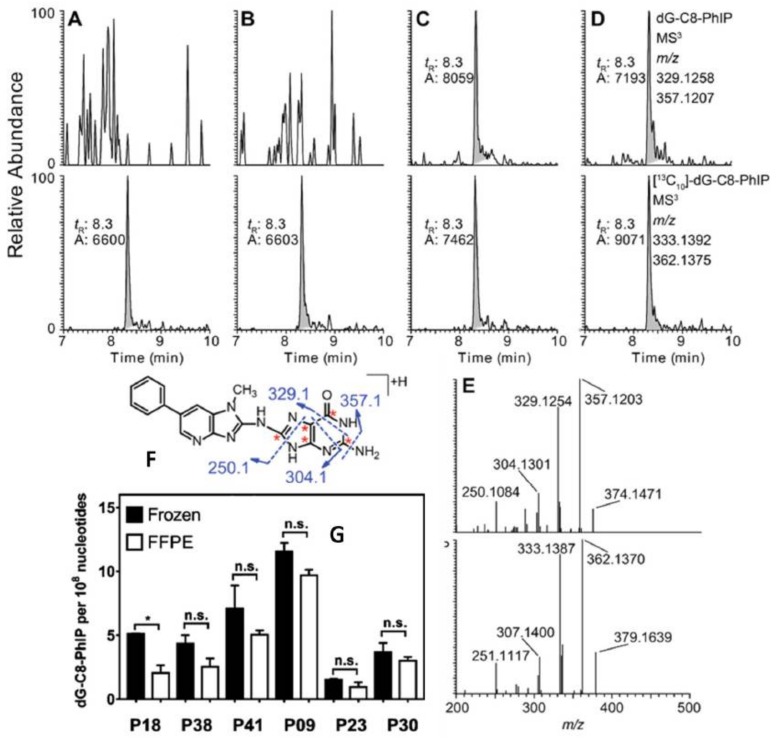
Extracted ion chromatograms of dG-C8-PhIP and ^13^C-labeled dG-C8-PhIP of DNA from fresh frozen and FFPE human prostate tissues at the MS^3^ scan stage. (**A**) Fresh frozen prostate and (**B**) paired FFPE block of a patient who was negative for dG-C8-PhIP; (**C**) fresh frozen prostate and (**D**) paired FFPE block of a patient who was positive dG-C8-PhIP at MS^3^ scan stage. The structure and proposed fragmentation mechanism of aglycone of dG-C8-PhIP are depicted fresh frozen prostate and (**D**) paired FFPE block of a patient who was positive for dG-C8-PhIP. (**E**) The product ion spectra at MS^3^ of unlabeled and ^13^C-labeled dG-C8-PhIP are shown. (**F**) Levels of dG-C8-PhIP in paired fresh frozen prostate and FFPE blocks of six patients are shown in (**G**). The levels of adducts are reported as adducts per 10^8^ nucleotides. * *p* < 0.05; n.s., statistically not significant. Reproduced with permission from [108]. Copyright ACS, 2017.

The recent improvements in sensitivity of mass spectrometry instrumentation has allowed us to use only 10 to 20 mg of tissue to screen for DNA adducts of environmental and dietary carcinogens in human biopsy samples [100,101,104]. We sought to determine if DNA extraction kits devoted to genomics, such as the FFPE miniprep kit from Zymo Research, could be employed to screen for DNA adducts in human FFPE biospecimens. We applied the method of DNA isolation to assay tissue section-cuts of human FFPE kidney specimens (1.5 cm^2^ × 10 µm) from the patients with upper urinary tract carcinoma, who were exposed to AA-I [79,104]. The levels of AA-I DNA adduct measured in FFPE tissues were comparable to those of matching frozen tissues (Figure 6). Some of these FFPE blocks had been stored at room temperature for four to nine years. This was the first report of quantitative measurement of a carcinogen DNA adduct in human FFPE tissue by mass spectrometry. We subsequently showed that DNA adducts of PhIP can be recovered in high yield from human FFPE prostate tissue blocks of prostate cancer patients stored at room temperature for at least 6 months (Figure 7) [101,108]. These findings show that FFPE tissues can be used to retrospectively screen for multiple classes of carcinogen DNA adducts. 

### 3.4. Rapid Throughput Method of DNA Extraction from FFPE Tissue

**Figure 8:**
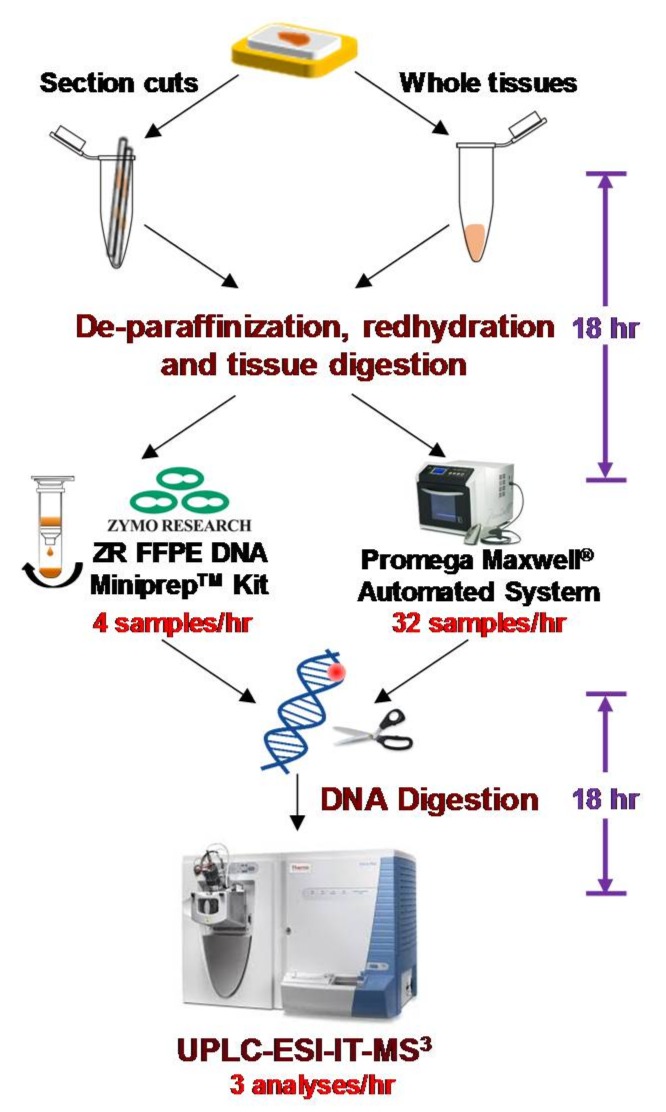
Scheme of FFPE tissue processing for DNA adduct measurements. DNA is extracted from section cuts or excised whole tissues by the FFPE Miniprep kit (Zymo Research) or Promega Maxwell^®^ automated system. After nuclease digestion, the DNA adducts are measured by UPLC-ESI-IT-MS^3^. The estimated times of the different processes are reported.

The method of DNA isolation from FFPE tissues employing the FFPE miniprep kit (Zymo Research) is robust; however, it is a manual and labor-intensive technique and cannot facilely process the large number of samples required for epidemiological studies. We developed a rapid throughput method of DNA isolation from FFPE tissue employing a semi-automated commercial DNA isolation system, Promega Maxwell**^®^** 16 MDx system, which is used for genomic research [108]. The system employs silica-magnetic beads technology for DNA isolation and can process 32 samples per hour compared to 4–6 samples per hour by the manual method. The DNA recovered from FFPE tissues using the Promega Maxwell**^®^** 16 MDx is fully digestible by nucleases [108]. The levels of dA-AL-I, dG-C8-4-ABP, and dG-C8-PhIP recovered from DNA of FFPE tissues extracted by rapid throughput method are comparable to those levels measured from DNA isolated by phenol-chloroform in matching frozen tissues, and in DNA of FFPE tissues isolated by the commercial manual Zymo kit [108]. With this advancement in DNA isolation technology, we believe that archived FFPE tissues can be used to screen for DNA adducts in large population studies. A scheme and the time of duration of the procedure to isolate DNA from FFPE section cuts or whole tissues, and ensuing chemical analysis by mass spectrometry, are depicted in Figure 8. The recovery of DNA from FFPE tissues and DNA digestion steps require overnight incubation with enzymes to achieve optimal digestion efficiency. The targeted and simultaneous quantification of a selected number of DNA adducts, by UPLC-ESI-IT-MS^3^, can be achieved in a 10 to 15 min run time.

### 3.5. Future Applications of DNA Adduct Measurements in Human Tissues

Although this review has focused on DNA adducts of environmental and dietary carcinogens, the measurements of DNA adducts of chemotherapeutic agents, such as platinum drugs and nitrogen mustards used to treat cancer, also can be measured in fresh frozen and FFPE tissues by mass spectrometry. Drugs that modify the structure of DNA and target cancer cells by interfering with DNA synthesis and cell replication often remain first line of medications used in cancer treatment. Thus, the efficacy of many anticancer drugs is thought to be linked to the levels of specific DNA adducts formed during drug treatment, and the quantitative measurements of the DNA adducts may be used as predictive markers in precision medicine to identify individuals who are most likely to benefit from treatment from those patients who may be less responsive to the therapy [109]. The assessment of DNA adducts of chemotherapeutic drugs and their cellular biological responses has been mostly performed in surrogate specimens, such as white blood cells or in vitro using cell lines rather than in the target cells or tumors, because of the invasiveness of biopsy sampling [109]. However, the exquisite sensitivity of current mass spectrometry instrumentation can allow for measurements that characterize the relationships between level of anticancer drug DNA adducts and pharmacodynamic response in patients using only 10 mg of tissue. As the sensitivity of MS instrumentation continues to improve, the amount of tissue specimen required for analysis will be further reduced, and the application of DNA adduct monitoring of chemotherapeutic drugs in clinical settings can be achieved.

The screening of DNA from FFPE tissues shows great promise to measure DNA adducts of multiple classes of carcinogens and anticancer drugs [37,55,105,109]. While most analyses have focused on one to several adducts, different types of MS scanning approaches are being developed to simultaneous scan for multiple types of DNA adducts in the field of DNA adductomics [63]. Triple quadrupole, quadrupole time-of-flight, ion trap or Orbitrap mass spectrometry instrumentation are being employed in DNA adductomics [55,58,62,63,110]. Our laboratory is developing unbiased non-targeted ion trap and Orbitrap scanning methods to screen for an array of DNA adducts in the human genome in a single assay [55,58]. Some of these adducts are expected to contribute to the tumor mutation burden [111].

A critical need is the development of accompanying informatic tools for data analysis and statistical tools to screen for covalent DNA damage. These scanning technologies and accompanying data analysis tools will provide a wealth of information about the exogenous and endogenous chemicals that damage the genome and may contribute to cancer risk. The implementation of FFPE tissues in DNA adduct biomarker discovery can provide the clues about the origin of human cancers for which an environmental exposure is suspected. 

## Figures and Tables

**Table 1:** Examples of DNA adducts detected in FFPE tissues.

Detection Methods	DNA Adducts Detected	Tissues	LOD (Per 10^8^ Nucleotides)	References
**IHC**	4-ABP-DNA	Human bladder	NR ^a^	[83]
	PAH-DNA	Human breast	NR ^a^	[84,85,92]
		Human esophagus	NR ^a^	[81,89]
		Human prostate	8	[90]
		Human cervix	20	[91]
		Human vulva	8	[47]
		Human placenta	20	[80]
	DMAB-DNA	Rat multiple tissues	NR ^a^	[96]
	PhIP-DNA	Human prostate tissue transplanted to mice	NR ^b^	[96]
		Rat multiple tissues	NR ^b^	[97]
	8-OHdG	Mouse pulmonary epithelial cells	NR ^a^	[102]
	Tamoxifen-DNA	Rat hepatocytes	10	[94]
**^32^P-postlabeling**	B[*a*]P-DNA, 2-AAF-DNA	Rat multiple tissues	NR ^c^	[103]
**LC-MS^3^**	dA-AL-I	Mouse liver and kidney, human kidney	0.1	[79,104]
	dG-C8-4-ABP/PhIP, dG-*N*^2^-BPDE, *O*^6^-Me-dG and *O*^6^-POB-dG	Rodent multiple tissues	0.2–0.5	[105]
**LC-HR-MS^2^**	dG-C8-PhIP	Human prostate	0.13	[101]
	dG-C8-4-ABP	Human bladder	0.2	[55]

^a^ Adduct levels were reported as relative nuclear stain intensity; ^b^ Adduct levels were reported as a percentage of positive cells; ^c^ LOD was reported in the citation, which was one per 10^10^ nucleotides employing 10 µg DNA. NR: Not reported.
